# A case of recurrent breast cancer with severe hepatic dysfunction: Integrating narrative-based medicine and clinical decision-making

**DOI:** 10.1017/S1478951526102454

**Published:** 2026-05-14

**Authors:** Kei Yamaguchi, Masahiro Ohara, Kazuo Matsuura, Hiroshi Ishiguro, Akihiko Osaki, Takao Takahashi, Hideki Onishi, Toshiaki Saeki

**Affiliations:** 1 Division of Breast Surgery, Saitama Medical Center, Saitama, Japan; 2 Department of Breast Oncology, Saitama Medical University International Medical Center, Saitama, Japan; 3 Department of Surgery, Maruyama Memorial General Hospital, Saitama, Japan; 4 Departments of Psycho-oncology, Saitama Medical University International Medical Center, Saitama, Japan

**Keywords:** Breast cancer, palliative care, chemotherapy, narrative-based medicine, shared decision-making

## Abstract

**Background:**

Patients with recurrent breast cancer and liver metastases complicated by hepatic failure have limited treatment options and poor prognoses. Narrative-based medicine (NBM) and shared decision-making (SDM) may support patient-centered decisions even in critical clinical situations

**Objectives:**

To describe the role of NBM and SDM in guiding treatment decisions for a patient with recurrent breast cancer and diffuse hepatic metastases associated with severe liver dysfunction.

**Methods:**

We present the case of a woman with recurrent breast cancer who developed hepatic failure caused by diffuse liver metastases. Repeated SDM discussions were conducted among the patient, her family, and a board-certified breast oncologist with certification in palliative care. Chemotherapy with eribulin was initiated together with intensive supportive care despite life-threatening organ failure.

**Conclusion:**

Following the initial onset of adverse effects, the patient’s liver function improved, allowing continuation of outpatient chemotherapy and fulfillment of her goal of spending meaningful time with family. The patient survived for approximately 5 months after treatment initiation.

**Significance of results:**

This case suggests that individualized care guided by NBM and SDM may support safe and goal-concordant treatment decisions, even near the end of life. Integration of oncologic and palliative expertise may help align medical interventions with patient values and preferences in complex clinical situations.

## Introduction

Narrative-based medicine (NBM), proposed in 1998, complements evidence-based medicine (EBM) and aids in realizing true patient-centered care (Suzuki and Takei 2025). Although interdisciplinary team-based care is essential, the complexity and specialization of oncology result in temporal and logistical constraints.

Herein, we present the case of a patient with recurrent breast cancer with life-threatening hepatic failure managed successfully with systemic therapy, including chemotherapy. Repeated shared decision-making (SDM) with a board-certified breast specialist, certified in palliative care, enabled the patient to choose active treatment despite critical illness, leading to an improved quality of life and prognosis.

## Case report

### Clinical course leading up to admission

The patient was a woman in her 50s who had previously undergone surgery for breast cancer on the left side. Her medical history included hypertension and myasthenia gravis with no known drug allergies.

**Figure 1.**
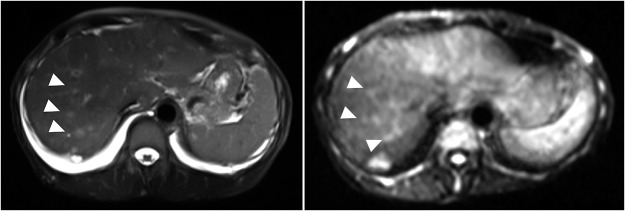
Axial T2-weighted and diffusion-weighted MRI examination of the liver. Multiple hyperintense areas are diffusely distributed throughout the liver parenchyma, as indicated by white triangles. These findings are consistent with occult, infiltrative liver metastases from breast cancer, lacking discrete nodular formation.

**Figure 2.**
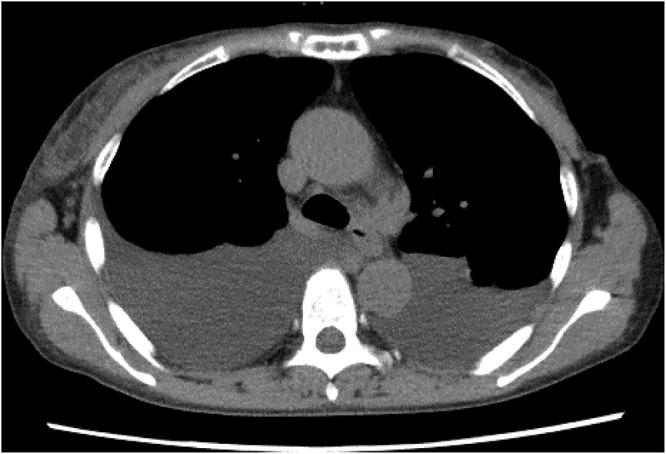
Chest computed tomography scan demonstrating large-volume bilateral pleural effusions. The effusions caused compression of the underlying lung parenchyma, contributing to respiratory compromise.

In June 2023, the patient developed recurrent disease with multiple liver and bone metastases, for which paclitaxel was initiated. By October of the same year, she presented with progressive fatigue, and her liver function deteriorated rapidly. Despite tumor shrinkage, drug-induced liver injury was suspected, and all anticancer agents and other potentially hepatotoxic drugs were discontinued. However, her liver function continued to deteriorate. She was referred to the Department of Gastroenterology and Hepatology, where imaging studies, including magnetic resonance imaging (MRI) (Figure 1), showed hepatic metastasis progression. Concurrently, worsening edema and malignant pleural and peritoneal effusions were observed. Chest computed tomography revealed pleural effusion (Figure 2), cytologically positive for malignancy. Elevated tumor markers supported disease progression.

A biopsy revealed adenocarcinoma, with immunohistochemistry showing estrogen receptor positivity of 2%, progesterone receptor negativity, and a human epidermal growth factor receptor 2 score of 1+. Subsequently, jaundice developed, along with further liver dysfunction.

At this stage, the clinical team considered 2 options: best supportive care (BSC) alone, given possible deterioration from further chemotherapy, or resumption of systemic treatment, given the potential benefits and risks. When BSC was presented as a treatment option, an SDM process was initiated at this point. The key issues included the limited prognosis, the uncertain survival benefit of chemotherapy, and the risk of further hepatic deterioration. The attending physician, a board-certified breast specialist with palliative care certification, led the process, coordinating a multidisciplinary clinical team that included medical oncologists, general oncology nurses, certified palliative care nurses, palliative care physicians, psycho-oncology specialists, clinical psychologists, pharmacists, and rehabilitation therapists. While the patient wished to avoid treatment-related suffering, she also expressed a strong desire to spend as much time as possible with her family, particularly to support her daughter emotionally. Through repeated discussions, the patient and her family reached a shared understanding of the goals of care, balancing life prolongation and quality of life. At first, both the patient and her family were greatly confused, and it was not easy for them to accept the options presented. To support their understanding, repeated SDM discussions were conducted to provide updated explanations of her condition and to reassess treatment goals as her clinical status evolved. These discussions involved the patient, her husband, and her daughter, as well as the primary physician, ward nurses, and, when appropriate, palliative care team members. Meetings were held once a week for approximately 1 hour, with additional sessions arranged as needed depending on clinical changes and the family’s availability. Through this structured, continuous process, the patient and her family gradually reached a shared understanding of the goals of care, balancing life prolongation and quality of life. We explained to the patient and family that the expected prognosis was limited, estimated at approximately 2–3 months, consistent with previous studies describing poor outcomes in patients with breast cancer and significant hepatic dysfunction (Mano et al. 2005). Both the option of no further systemic therapy and the option of chemotherapy were presented in parallel. For chemotherapy, multiple regimens, including eribulin, trastuzumab deruxtecan, and oral 5-fluorouracil, were explained side by side. We clearly communicated that, despite treatment, her condition might worsen, potentially leading to life-threatening complications. Although eribulin treatment causes hepatotoxicity, particularly in hepatic impairment, it is a feasible option with appropriate dose modification. In our case, considering the preserved liver function (Child-Pugh B), eribulin was selected as palliative systemic therapy after SDM, balancing the potential benefits and risks. Pivotal clinical trials (Studies 301 and 305) provided detailed guidance on dose adjustments based on Child-Pugh classification and liver function tests (Macpherson et al. 2021). The patient expressed concern about adverse effects, particularly nausea, and wished to minimize both physical toxicity and treatment time burden. Thus, eribulin chemotherapy was initiated under close monitoring. Since recurrence, repeated and thorough discussions with the patient and her family ensured a shared understanding of her condition. The patient lived with her husband and daughters. Following the diagnosis of recurrence, the patient’s daughter experienced her first depressive episode, which was considered to have been triggered by psychological distress related to her mother’s illness. The daughter received outpatient care from the same psycho-oncology physician who was also involved in the patient’s care. Both the patient and her husband had a good understanding of the disease and appeared to have accepted the inevitability of death from breast cancer. The psycho-oncology team was aware that the patient was concerned about her daughter’s depression when considering chemotherapy. However, they did not intervene in the treatment decision itself and respected the autonomy of the patient and family in the SDM process. While the patient hoped to avoid suffering from the side effects of chemotherapy, she prioritized spending more time with her family, believing that it could help her daughter prepare emotionally. Ultimately, she opted for chemotherapy, hoping to extend her life by a few months to allow her daughter time to come to terms with her terminal illness. Given the disease severity and need for close symptom management, including control of pleural and peritoneal effusions, chemotherapy was planned under inpatient care.

Before starting treatment, detailed discussions were held with the patient, her husband, and daughter regarding the potential for severe adverse events and that disease progression could deteriorate the performance status or make it impossible to return home. The team emphasized that treatment should be pursued, and maximal supportive care should be provided in parallel, including drainage of ascites and pleural effusion, to maintain quality of life.

During the previous hospitalization, immediately after recurrence, the patient experienced significant distress due to the large volume of information and the need to consider discharge and end-of-life care in a short time. In particular, being asked to decide on a place of care or death created pressure. To reduce anxiety, the physician intentionally avoided establishing a care location in advance, instead organizing home medical care support and ensuring that the patient could be readmitted if her condition worsened.

### Clinical course during hospitalization

Upon admission, laboratory tests revealed hypoalbuminemia (albumin, 2.7 g/dL) and elevated lactate dehydrogenase levels (570 U/L), indicating liver dysfunction. Liver enzyme levels were elevated: aspartate aminotransferase (AST), 212 U/L and alanine aminotransferase (ALT), 49 U/L. The total bilirubin level increased to 3.7 mg/dL, consistent with jaundice. Coagulation studies showed a slightly prolonged prothrombin time (PT-international normalized ratio, 1.16; PT, 71%). Tumor markers were markedly elevated: carcinoembryonic antigen, 3975 ng/mL and cancer antigen 15-3, 542 ng/mL (Table 1).

**Table 1.** Laboratory findings on admission

Item	Result	Unit	Item	Result	Unit
WBC	5547	/μL	AST	*212	U/L
Hb	15.6	g/dL	ALT	*49	U/L
PLT	184 × 10^3^	/μL	T.Bil	*3.7	U/L
ALB	*2.7	g/dL	D.Bil	*2.6	U/L
Cre	0.49	mg/dL	CRP	0.681	mg/dL
BUN	12.9	mg/dL	PT-INR	*1.16	
Na	136	mmol/L	PT%	*71	%
K	3.7	mmol/L	APTT	32.9	Sec
LDH	*570	U/L	CEA	*3975	ng/mL
CK	*587	U/L	CA15-3	*542	ng/mL

WBC, white blood cell count; Hb, hemoglobin; PLT, platelet count; AST, aspartate aminotransferase; ALT, alanine aminotransferase; T.Bil, total bilirubin; D.Bil, direct bilirubin; ALB, albumin; Cre, creatinine; BUN, blood urea nitrogen; CRP, C-reactive protein; PT-INR, prothrombin time-international normalized ratio; PT%, prothrombin activity; APTT, activated partial thromboplastin time; LDH, lactate dehydrogenase; CK, creatine kinase; CEA, carcinoembryonic antigen; CA15-3, cancer antigen 15-3.

* indicates abnormal values.

On hospital day 1, eribulin 0.7 mg/m^2^ was initiated. By days 2–3, the patient experienced grade 2 nausea and vomiting, which were managed symptomatically; however, on day 3, worsening edema and ascites developed, along with rising liver enzyme and bilirubin levels. These clinical changes prompted reconsideration of whether treatment should be continued within the framework of SDM. The multidisciplinary team reassessed her clinical status and reinforced supportive care measures while openly discussing the risks and uncertainties with the patient and her family. Although the patient expressed concern about further deterioration, she reaffirmed her wish to proceed with treatment after discussion, prioritizing the possibility of extending meaningful time with her family. As her condition worsened, her prognosis and overall goals of care were revisited with the patient and her family, and symptomatic management was intensified. The ascitic fluid was transudative, suggesting portal hypertension secondary to liver metastases. On day 7, tolvaptan 7.5 mg was added. Owing to worsening physical symptoms, the patient requested postponing the next eribulin dose.

By day 8, the liver enzyme levels began to improve, suggesting a possible therapeutic effect. The patient initially hesitated to continue therapy because of the discomfort caused by nausea and fatigue. However, after further discussion with the physician, including plans to intensify antiemetic prophylaxis, she decided to proceed. On day 18, eribulin was re-administered, and significant adverse events were avoided with strengthened antiemetic support. By day 22, the patient’s condition had stabilized sufficiently for discharge.

**Figure 3.**
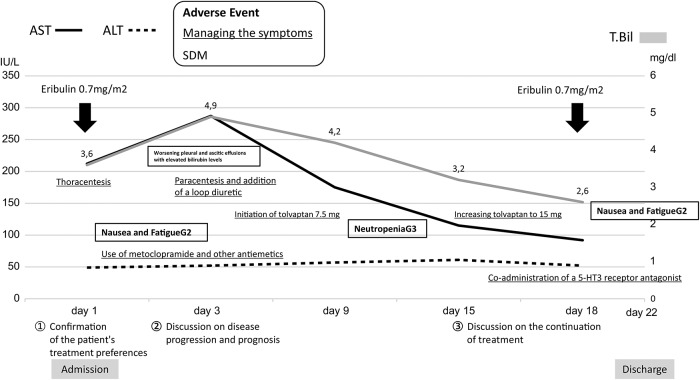
Clinical course after admission, showing temporal changes in liver function markers. The *Y*-axis represents serum levels of AST, ALT (left), and total bilirubin (T.Bil; right), while the *X*-axis denotes days from admission. Eribulin (0.7 mg/m^2^) was administered on days 1 and 15 (black arrows). After initial treatment, total bilirubin rose transiently with worsening ascites and pleural effusion, followed by gradual improvement. Interventions, including thoracentesis, paracentesis, and tolvaptan dose adjustments, are shown. Adverse events, such as fatigue, dyspnea, and grade 3 neutropenia, are in bold. Symptom management points are underlined, and shared decision-making (SDM) points are shown by shading.

At discharge, SDM guided the establishment of an end-of-life care plan in collaboration with a local hospital and home-visit physician. The plan allowed flexibility between home and hospital care without forcing a final decision, and the patient could be readmitted at any time if her condition worsened. The patient and her family preferred home care to maximize time spent together, and concerns about caregiving burden were addressed through support from the home medical team. The clinical course is summarized in Fig. 3, showing changes in total bilirubin levels and liver function over time.

### Post-discharge course

**Figure 4.**
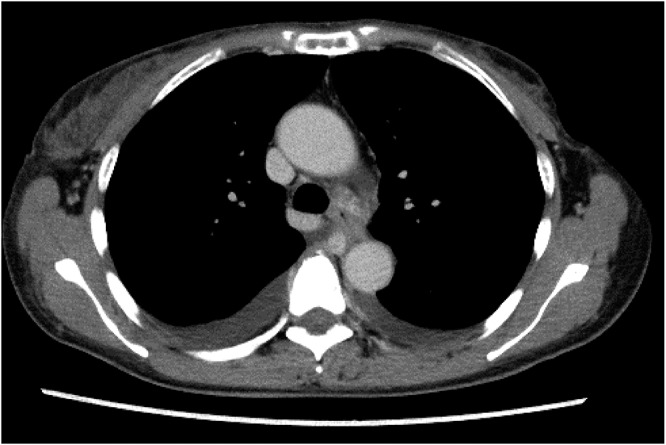
Chest computed tomography scan showing continued reduction in bilateral pleural effusions.

After discharge, chemotherapy was continued as an outpatient for approximately 4 months, during which liver function remained stable and without worsening effusions or edema (Figure 4). Remarkably, the patient could travel with her family, a goal that seemed impossible during hospitalization, and neither she nor her family regretted the decision to pursue treatment.

Approximately 5 months after discharge, the disease progressed with worsening liver metastases, and she died peacefully at a local hospital. Adequate communication with both the medical team and family allowed the patient to share meaningful time with her loved ones, helping her husband and daughter maintain emotional stability and continue their daily lives, including their work.

## Discussion

The prognosis for breast cancer with liver metastases remains limited, with reported median overall survival ranging from approximately 16 to 27 months in recent cohorts, and 5-year survival rates below 10% in some studies (Pentheroudakis et al. 2006; Ji et al. 2021; Tian et al. 2021). Diffuse liver metastases usually present as nodular lesions; however, occasionally, extensive infiltration becomes radiologically occult, leading to acute liver failure and consequently a markedly poor prognosis (Allison et al. 2004). This is more common in hematologic malignancies; reports in solid tumors, such as breast cancer, are rare (Hanamornroongruang and Sangchay 2013). The MRI findings in our case supported the diagnosis of diffuse progression, similar to carcinomatous cirrhosis (Hoshina et al. 2021).

Eribulin was selected because of its proven efficacy, short infusion time, and manageable toxicity (Cortes et al. 2011). Although the patient experienced grade 2 fatigue, nausea, and grade 3 neutropenia, treatment was safely continued after dose adjustment for liver dysfunction.

Modern oncology increasingly relies on specialized multidisciplinary teams, but time constraints and fragmented roles can lead to communication gaps. SDM addresses this by fostering dialogue and respecting patient autonomy, rather than merely presenting options (Kandabashi 1984; Charles et al. 1997; Elwyn et al. 2012). NBM, by framing illness as a patient’s story and integrating it with medical expertise, complements EBM and facilitates the practice of SDM (Charon 2001). EBM, defined as “the conscientious, explicit, and judicious use of the current best evidence to make decisions about the care of individual patients” (Sackett et al. 1996), is not a universal or infallible methodology, but a framework applied to each patient. Understanding the EBM–NBM relationship fosters SDM, ultimately enhancing patient and family satisfaction (Greenhalgh 1999).

In this case, repeated conversations helped the patient, initially hesitant about further treatment, reconsider her personal narrative and family context. This approach allowed chemotherapy along with continued supportive care, resulting in prolonged survival and greater patient and family satisfaction. Although prolonged survival was achieved, the sustained practice of SDM with the involvement of a multidisciplinary team also held important significance in the context of end-of-life care.

The attending physician’s role remains central even in highly specialized modern care. For truly patient-centered care, oncologists should integrate palliative care knowledge and respect each patient’s narrative along with evidence-based practice.
